# Mutations in the Non-Catalytic Subunit Dpb2 of DNA Polymerase Epsilon Affect the Nrm1 Branch of the DNA Replication Checkpoint

**DOI:** 10.1371/journal.pgen.1006572

**Published:** 2017-01-20

**Authors:** Michał Dmowski, Justyna Rudzka, Judith L. Campbell, Piotr Jonczyk, Iwona J. Fijałkowska

**Affiliations:** 1 Institute of Biochemistry and Biophysics, Polish Academy of Sciences Pawińskiego 5a, Warsaw, POLAND; 2 Braun Laboratories, California Institute of Technology, Pasadena, CA, United States of America; University of Minnesota Twin Cities, UNITED STATES

## Abstract

To preserve genome integrity, the S-phase checkpoint senses damaged DNA or nucleotide depletion and when necessary, arrests replication progression and delays cell division. Previous studies, based on two *pol2* mutants have suggested the involvement of DNA polymerase epsilon (Pol ε) in sensing DNA replication accuracy in *Saccharomyces cerevisiae*. Here we have studied the involvement of Pol ε in sensing proper progression of DNA replication, using a mutant in *DPB2*, the gene coding for a non-catalytic subunit of Pol ε. Under genotoxic conditions, the *dpb2-103* cells progress through S phase faster than wild-type cells. Moreover, the Nrm1-dependent branch of the checkpoint, which regulates the expression of many replication checkpoint genes, is impaired in *dpb2-103* cells. Finally, deletion of *DDC1* in the *dpb2-103* mutant is lethal supporting a model of strand-specific activation of the replication checkpoint. This lethality is suppressed by *NRM1* deletion. We postulate that improper activation of the Nrm1-branch may explain inefficient replication checkpoint activation in Pol ε mutants.

## Author Summary

The viability of living organisms depends on the integrity of their genomes. Each cell has to constantly monitor DNA replication and coordinate it with cell division to avoid genomic instability. This is achieved through pathways known as cell cycle checkpoints. Therefore, upon replication perturbation, DNA synthesis slows down and cell division is delayed. For that, a specific signal is induced and propagated through a mechanism that have already been identified but still need investigations. We have isolated a mutated form of Dpb2, the essential subunit of DNA polymerase epsilon (Pol ε) holoenzyme. This mutated form of Pol ε impairs proper activation of the cellular response to replication stress. We show that yeast cells with mutations in the *DPB2* gene fail to activate the Nrm1-regulated branch of the checkpoint, which controls numerous genes expressed in response to replication stress. Moreover, our results support the model of parallel activation of replication checkpoint from the leading and lagging DNA strands. This strongly suggests that Pol ε, the leading strand replicase, is involved in replication checkpoint activation from this strand. Our results contribute to the understanding of mechanisms of cellular response to replication stress, which are necessary to preserve genome stability.

## Introduction

DNA integrity of living organisms is affected by perturbations that induce replication stress including nucleotide depletion or collision with lesions encountered in DNA exposed to alkylating agents [1]. Therefore, each cell must constantly monitor its genome integrity and coordinate DNA replication with cell division in order to avoid genetic instability [2]. Cell cycle checkpoints that monitor the accuracy of each phase of the cycle play crucial role in this control. The replication checkpoint monitors DNA duplication, and when activated, regulates transcription of specific genes, arrests replication progression, stabilizes replication forks, increases the dNTP pool, suppresses late-origin firing, delays cell division and finally restarts DNA synthesis after removal of replication stress [3–10]. It also prevents homologous recombination (HR) at double strand breaks (DSB) and stressed replication forks during S phase, presumably by blocking DNA ressection, to prevent genetic instability [11,12].

Checkpoint mechanisms encompass many proteins that act as sensors, mediators and effectors in a cascade of phosphorylation events [13]. In the first step, uncoupling of helicase and polymerase activities, unsynchronized leading and lagging strand replication or replication fork collapse result in accumulation of ssDNA [14,15]. After an activation threshold is reached [16], large stretches of RPA-coated ssDNA recruit the apical protein kinase Mec1 bound to Ddc2 [17]. Then, the Ddc1 subunit of the 9-1-1 sensor checkpoint clamp (Ddc1-Rad17-Mec3 in *Saccharomyces cerevisiae*) is recruited to the ds-ssDNA junctions and activates the signaling network [18]. The checkpoint response is completely dependent on the 9-1-1 complex in G1 phase while in G2 Dpb11 is also involved in this process [19]. In the S-phase, multiple factors are needed to trigger checkpoint activation including Dna2 in addition to Ddc1, Dpb11 [20–22] reviewed in [13,23]. It has been shown that a *ddc1Δ dpb11-1* double mutant is partially defective in phosphorylation of the checkpoint effector kinase, Rad53 [20,24], indicating that there is an additional S-phase checkpoint activation pathway. Since Dna2 is probably involved in this additional activation mechanism, in the triple *dpb11Δ ddc1Δ dna2Δ* mutant only negligible phosphorylation of Rad53 was detected [21]. Finally, there is also evidence that DNA polymerase epsilon (Pol ε) is involved in the 9-1-1 independent activation pathway (Dpb11 recruitment to stalled replication forks) [25] suggesting separation of replication stress sensors on the leading and lagging DNA strands [20,26].

Upon checkpoint activation, the phosphorylated signaling kinase Mec1, transmits the signal to the downstream effector kinase Rad53 [27]. Its activation during replication stress is facilitated by checkpoint mediator protein Mrc1 [28,29] which promotes Mec1-Rad53 interactions [30]. Importantly, both Mec1 and Rad53 are essential genes in *S*. *cerevisiae* while not in *Schizosaccharomyces pombe* [31]. Rad53-dependent control of the replication stress response is divided into two branches: (i) the well-characterized Dun1-Crt1 pathway, also called DNA damage response (DDR) branch [32,33], which mainly up-regulates the dNTP pool, and (ii) the Nrm1-MBF pathway, also called the G_1_/S cell cycle (CC) branch [34,35], which up-regulates dozens of genes involved in many processes e.g., *TOS4*, *TOS2*, *MCD1*, *CDC21* [36].

Pol ε is one of the major replicative polymerases that generally replicates the leading DNA strand while DNA polymerase delta (Pol δ) replicates the lagging strand [37–40]. Recently, an *in vitro* study of a reconstituted replisome has shown that Pol ε is targeted to the leading strand by the CMG complex (Cdc45, Mcm2-7 and GINS) while Pol δ is targeted to the lagging strand by PCNA (proliferating cell nuclear antigen) [41]. Moreover, a chromatin immunoprecipitation based method (eSPAN) was used to demonstrate the same strand bias patterns of Pol δ and Pol ε [42].

Pol ε is composed of the catalytic Pol2 subunit and three non-catalytic subunits Dpb2, Dpb3 and Dpb4 [43–45], for review see [46,47]. Dpb3 and Dpb4 subunits are involved in stabilization of Pol ε interaction with DNA, and their deletion affects replication fidelity [48]. Pol2 and Dpb2 subunits are essential in yeast, although deletion of the N-terminal polymerase catalytic domain of Pol2 gives viable cells [49,50]. In contrast, its C-terminal half is necessary and sufficient to support growth and is involved in both interaction with the Dpb2 subunit and S-phase checkpoint activation [50–52]. The interaction of Dpb2 subunit with Psf1, a subunit of the GINS complex, is important for the CMG complex assembly. Therefore, Dpb2 is involved in initiation of DNA replication but also links Pol ε to the CMG complex during elongation [53–57]. Finally, Pol ε –GINS interaction enables the preferential recruitment of Pol ε over Pol δ to the leading strand [41].

The *dpb2* mutants isolated in our laboratory demonstrate temperature-sensitivity and an increased number of replication errors (MMR-dependent mutator phenotype) [58,59]. In these mutator strains, Pol ζ participates in DNA replication more often although the mutator phenotype of *dpb2* mutants results not only from this error-prone TLS polymerase activity [60]. Moreover, these Dpb2 mutants are impaired in interaction with Pol2 and the GINS subunits Psf1 and Psf3 [56,58,61] which may result in increased participation of Pol δ on the leading strand and be partially responsible for the mutator phenotype [61].

In this work, we investigate the involvement of the Dpb2 subunit of Pol ε in triggering the response to replication stress. For this purpose, we use the *dpb2-103* mutant carrying T342I S343F T345I P347S P348S substitutions, isolated in our laboratory [58]. We found that this mutant demonstrates phenotypes characteristic for replication checkpoint mutants. The *dpb2-103* cells are sensitive to MMS (methyl methanesulfonate) and HU (hydroxyurea), and fail to delay cell cycle progression when treated with these agents. Although, *dpb2-103* cells undergo checkpoint-induced Rad53 phosphorylation, they cannot properly activate the Nrm1/MBF branch of downstream response. Finally, we observed a lethal effect of *dpb2-103* mutation combined with *ddc1Δ*. We propose that the observed synergy suggests independent roles in checkpoint activation and that 9-1-1 may recognize damage on the lagging strand while *dpb2*, as a subunit of Pol ε, acts on the leading strand.

## Results

### The *dpb2-103* mutant demonstrates an S-phase checkpoint deficiency phenotype

Studies of the replication stress checkpoint have suggested the involvement of the catalytic subunit of DNA polymerase epsilon, Pol2, in checkpoint activation [62,63]. Later, it was suggested that Dpb2, the essential non-catalytic subunit of Pol ε interacts with Mrc1, the checkpoint mediator, and that thus Dpb2 may also be involved in activation of the S-phase checkpoint through modulation of Pol2-Mrc1 interactions [64]. Dpb2 variants that contribute to a spontaneous mutator phenotype have been analyzed in our laboratory for many years [58–61].

**Fig 1:**
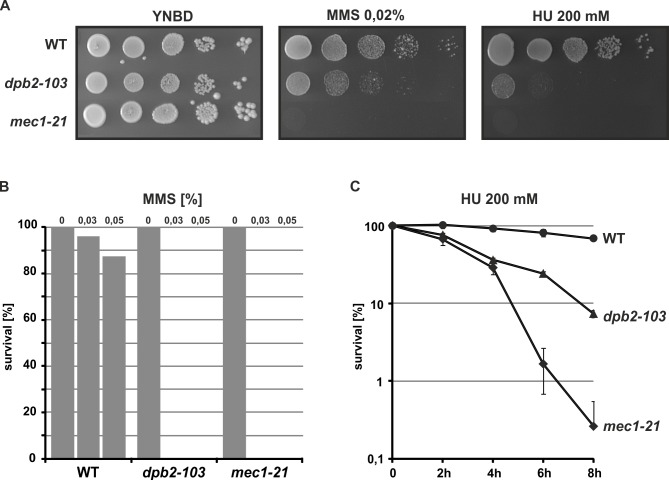
*dpb2-103* mutation affects yeast viability under genotoxic or replication stress. (**A)** Cultures of indicated strains were grown exponentially, serially diluted and spotted on YNBD supplemented with MMS or HU. Plates were incubated at 23°C for 5 days. (**B)** Log-phase cultures of yeast strains were appropriately diluted and plated on YNBD medium without MMS or supplemented with 0,03% or 0,05% MMS. Plates were incubated at 23°C for 5 days. (**C)** Log-phase cultures of yeast strains were supplemented with HU to final concentration 200 mM. Samples were collected at 2 hours intervals, plated on YNBD medium, and incubated at 23°C for 5 days.

Replication stress can be generated either by nucleotide depletion using HU or by blocking replication due to fork collision with MMS-generated DNA lesions, which are detected only during replication [1,65]. To determine whether Dpb2 protein is involved in proper execution of replication checkpoint, first we analyzed the sensitivity of yeast cells with the *dpb2-103* allele to the genotoxic agent methyl methanesulfonate (MMS) or to hydroxyurea (HU), the ribonucleotide reductase inhibitor (**Fig 1**). When compared to wild type cells, those with the *dpb2-103* allele demonstrate increased sensitivity to both MMS and HU, although these cells were not as sensitive as the canonical S-phase checkpoint deficient mutant *mec1-21* (**Fig 1A, 1B and 1C**).

**Fig 2:**
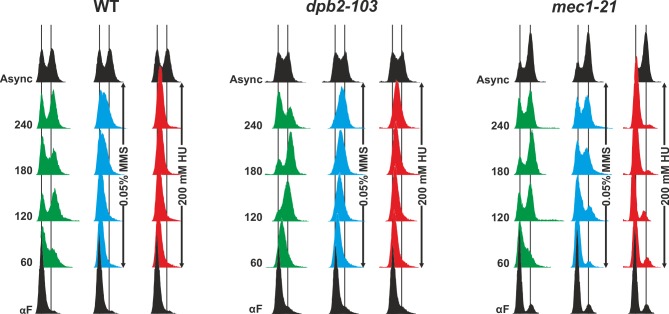
Functional *DPB2* is required to prevent cell cycle progression under genotoxic or replication stress. Cultures of indicated strains were synchronized in G1 and released from α-factor in YNBD (green), YNBD with 0,05% MMS (blue) or YNBD with 200 mM HU (red). Samples were taken every 60 minutes. For each strain, the DNA content was monitored by flow cytometry.

Yeast cells challenged with genotoxic or replication stress activate the checkpoint and delay their cell cycle progression. Slowing down the progression through S-phase gives more time to complete perturbed DNA replication and may result from inhibition of dormant or late origin firing [1,3,47] or inhibition of replication elongation [66]. This delay can be observed by flow cytometry analysis of DNA synthesis progression in the population of yeast synchronized in G1 and released under specific conditions [9,67,68]. We synchronized *dpb2-103* mutant yeast cells with α-factor and released them from G1 in the absence or presence of MMS or HU. Then, we performed a flow cytometry analysis of DNA content to monitor G1-S-G2 transitions. Under MMS treatment in minimal media at 23°C, *dpb2-103* cells reached the 2C DNA content after 240 min while wild-type cells remained at the G1-S transition after the same time in the same conditions. (**Fig 2**). Under HU treatment *dpb2-103* cells entered S-phase very slowly, whereas wild-type cells remained blocked in G1 phase. These results demonstrate that, similarly to the *mec1-21* checkpoint mutant, *dpb2-103* cells are defective in delaying cell cycle progression and DNA synthesis when challenged with replication stress.

The checkpoint-induced delay of cell cycle progression in cells exposed to HU enables replication fork stabilization and DNA synthesis restart after release from replication stress. Therefore, we synchronized *dpb2-103* cells in G1, and released them in the presence of HU. After 90 minutes, we washed out HU and shifted cells into fresh medium. Unexpectedly, unlike the control strain *mec1-21* cells, the *dpb2-103* cells were able to restart DNA synthesis after release from HU (**S1 Fig**). This result shows that the *dpb2-103* mutant retains partial S-phase checkpoint activity, perhaps due to 9-1-1 checkpoint clamp sensing from the lagging strand.

### Deletion of *DDC1* in *dpb2-103* is lethal

**Fig 3:**
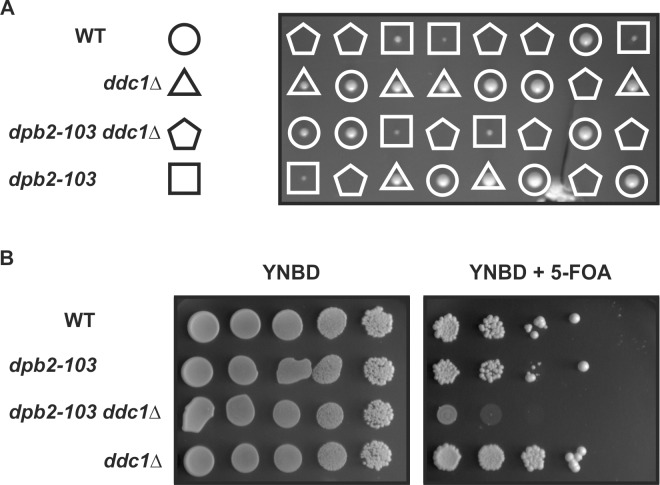
Synthetic lethality *dpb2-103 ddc1*Δ cells. **(A)** Tetrad analysis of a heterozygous *dpb2-103*/*DPB2*, *ddc1Δ*/*DDC1* strain. **(B)** Indicated strains with plasmid pMJDPB2 (source of *DPB2* allele) were grown exponentially, serially diluted and spotted on YNBD or YNBD supplemented with 5-FOA.

Previous work has suggested the involvement of the leading strand DNA polymerase ε in replication checkpoint activation [62,69]. At the same time, the 9-1-1 (Ddc1-Rad17-Mec3) complex has been proposed to be involved in sensing lagging strand replicative stress [20]. If the 9-1-1 checkpoint clamp and Pol ε act in parallel, strand-specific pathways to induce the response to replication stress, one can expect reduced ability to induce the checkpoint in the double mutant. To test whether Dpb2 is the Pol ε subunit involved in inducing the leading strand pathway of the replication stress checkpoint, we decided to introduce a *DDC1* deletion in the *dpb2-103* cells. Interestingly, the attempts to substitute *DDC1* with nourseothricin resistance cassette (*NAT1*) were unsuccessful. Similarly, the dissection of tetrads obtained from a *DPB2/dpb2-103 DDC1/ddc1Δ* strain (**Fig 3A**) failed to generate *dpb2-103 ddc1Δ* cells, suggesting that the double mutant *dpb2-103 ddc1Δ* is inviable. In order to verify this, we attempted to introduce a *DDC1* deletion into *dpb2-103* cells carrying the pMJDPB2 plasmid [58] that provides Dpb2 protein. Transformants obtained in this experiment were cultured and serial dilutions were plated on YNBD medium and YNBD supplemented with 5-FOA to obtain plasmid-free clones. As expected, in contrast to the wild-type, *dpb2-103* and *ddc1Δ* strains the *dpb2-103 ddc1Δ* cells became inviable after plasmid loss (**Fig 3B**) supporting the conclusion that the double mutant phenotype is lethal.

The S-phase checkpoint induction deficiency of canonical *mec1* or *rad53* mutants is rescued by increasing dNTP formation by deletion of the ribonucleotide reductase inhibitor gene *SML1* [4,70]. Therefore, we attempted to obtain *dpb2-103 ddc1Δ sml1Δ* cells through tetrad dissection of an appropriate heterozygous strain. However, after prolonged incubation, we obtained only small colonies of inviable double or triple mutants (**S2 Fig**). These results demonstrate that the *sml1Δ* (increased dNTP level) does not rescue lethality of *dpb2-103 ddc1Δ* cells. Together, these results demonstrate synthetic lethality of the *dpb2-103* mutation combined with deletion of the *DDC1* gene.

### The Dun1 / Crt1 pathway is properly activated in *dpb2-103* cells

Checkpoint activation in the S-phase induces a cascade of phosphorylation events. To test the stage at which checkpoint activation fails in *dpb2-103* cells, first we analyzed activation of the checkpoint kinase Rad53. [27,71]. We compared the phosphorylation of Rad53 in *dpb2-103* cells after MMS or HU treatment to the Rad53 status in checkpoint defective *mec1-21* and *pol2-12* cells. Migration of the phosphorylated form of Rad53 in polyacrylamide gels is retarded compared to unmodified Rad53. In *dpb2-103* cells, after MMS or HU treatment during 180 minutes, phosphorylated Rad53 was detected (**S3 Fig**). As expected, in the checkpoint defective control strain *mec1-21*, after either MMS or HU treatment no Rad53 phosphorylated form was observed. In *pol2-12* cells Rad53 was phosphorylated after HU treatment and residual phosphorylation was observed under MMS-induced genotoxic stress (**S3 Fig**). These observations suggest that although the *dpb2-103* mutant seems to be impaired in S-phase checkpoint activation, the checkpoint kinase Rad53 is phosphorylated. However, it is not clear whether the protein is phosphorylated properly and the downstream signal amplified and propagated correctly.

Rad53-dependent phosphorylation of Dun1 (*DNA-damage un-inducible*) up-regulates the dNTP pool, primarily through two mechanisms. First, Dun1 phosphorylates and thus inhibits the Crt1 (*constitutive RNR transcription*) repressor which regulates a small part of the checkpoint-dependent transcriptional response i.e. the *RNR2*, *RNR3* and *RNR4* genes which encode subunits of the ribonucleotide reductase RNR [32,72]. Crt1 also represses the expression of gene *HUG1* (*hydroxyurea and UV and gammaradiation induced*) whose product inhibits RNR through binding the Rnr2 subunit [73,74]. In parallel, Dun1 phosphorylates and promotes degradation of Sml1, the inhibitor of RNR [4,75] Moreover, Dun1-dependent phosphorylation of Dif1 (*damage-regulated import facilitator 1*), results in inactivation of the nuclear import of the Rnr2 and Rnr4 subunits of RNR, resulting in their cytoplasmic localization [76,77].

**Fig 4:**
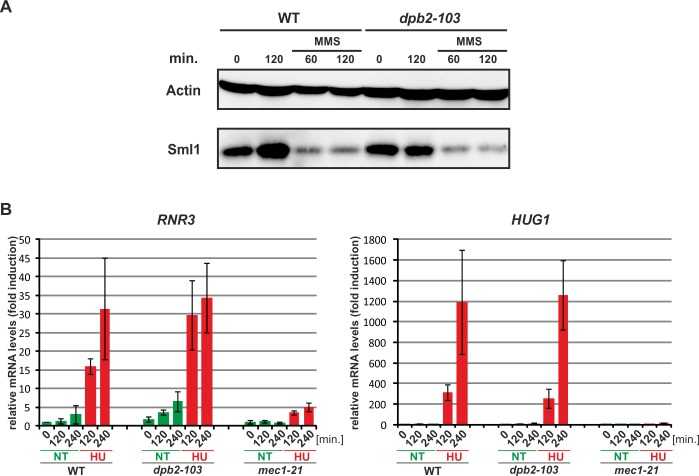
The Crt1/Dun1 pathway of the replication stress checkpoint is activated in *dpb2-103* cells under MMS or HU treatment. **(A)** Western-Blot detection of Sml1 degradation. Extracts from WT or *dpb2-103* yeast cells treated with 0,05% MMS were analyzed. **(B)** Quantitative RT-PCR analysis of *RNR3* and *HUG1* transcripts in yeast cells released from G1-arrest in YNBD (green) or YNBD with 200 mM HU (red). Transcript levels were analyzed after 120 and 240 minutes of growth and normnalized to wild-type G1-synchronized cells.

Therefore, to test whether in the *dpb2-103* cells the checkpoint was interrupted downstream of Rad53, we analyzed the degradation of Sml1 and induction of *RNR3* and *HUG1* genes. The amount of the Sml1 protein was analyzed immunologically in extracts from cells treated with MMS for 60 or 120 minutes and compared with untreated cells. In wild-type cells, under genotoxic stress Sml1 is degraded after 60 minutes. Similar results were observed for *dpb2-103* cells treated with MMS (**Fig 4A**). Next, using quantitative RT-PCR we analyzed the expression of the ribonucleotide reductase gene *RNR3* and gene *HUG1* encoding the RNR inhibitor [73,74]. Expression of these two genes is upregulated by the Dun1-Crt1 branch of replication stress response. Wild-type or *dpb2-103* cells were synchronized in G1 and released into S-phase in the presence of 200 mM HU. The amount of *RNR3* or *HUG1* transcripts was normalized to wild-type untreated cells. After 120 and 240 minutes of HU treatment, the *RNR3* and *HUG1* expression levels in *dpb2-103* were similar to those observed in wild-type cells under the same treatment (**Fig 4B**). It is noteworthy, that the *RNR3* mRNA levels at the time of release from G1 and after 120 or 240 from release into S-phase were about 2-fold higher than in wild-type cells. In the control experiment, in *mec1-21* cells, induction of *RNR3* or *HUG1* was not observed. These results demonstrate that the *dpb2-103* cells activate the Dun1-Crt1 branch in response to HU or MMS induced replication stress.

### The Nrm1 / MBF pathway is not activated properly in *dpb2-103* cells

**Fig 5:**
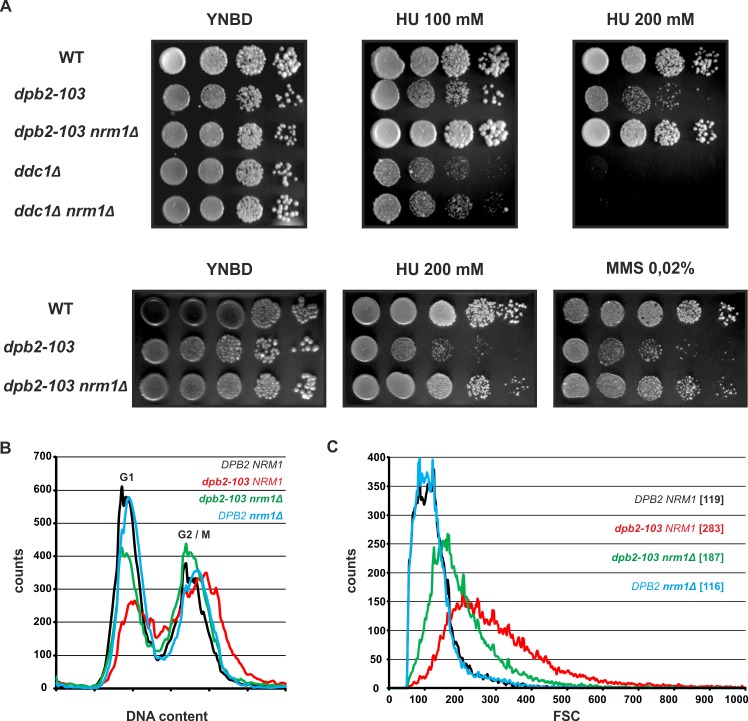
The MBF pathway of the replication stress checkpoint is not activated (derepressed) properly in *dpb2-103* cells under MMS or HU treatment. **(A)** Deletion of *NRM1* (coding for the MBF repressor) rescues *dpb2-103* but not *ddc1Δ-*mediated MMS or HU sensitivity. Cultures of indicated strains were grown exponentially, serially diluted and spotted on YNBD supplemented with MMS or HU. **(B)** The abnormal progression of replication and **(C)** the large-cell phenotype of *dpb2-103* cells are partialy rescued by *NRM1* deletion. Asynchronous cells were analysed by flow cytometry to evaluate DNA content (FL2) and cell size (FSC, forward scatter). FSC median values are given in brackets.

The replication checkpoint pathway downstream of Rad53 also encompasses a second branch, parallel to the Dun1/Crt1, i.e. the Nrm1/MBF branch. The Nrm1 co-repressor (*negative regulator of MBF targets 1*) together with the MBF (*MluI-binding factor*) repressor complex recognize the MCB (*MluI cell-cycle box*) DNA sequence in promoter regions of dozens of genes to repress transcription upon exit from G1 phase. Under replication stress, Rad53-mediated phosphorylation of the Nrm1 repressor prevents its binding to MBF promoters and allows upregulation of a set of MCB G1/S transition genes [34,35,78–83]. As a consequence, deletion of *NRM1* and expression of MCB genes increases cell survival of checkpoint-deficient *rad53Δ* or *mec1Δ* yeast cells challenged with replication stress [78,79]. Therefore, we hypothesized that upregulation of G1/S transition genes would also rescue *dpb2-103* sensitivity to replication stress. We saw that indeed *nrm1Δ* bypasses the sensitivity of *dpb2-103* cells in the presence of HU or MMS (**Fig 5A**). Interestingly, this was not the case for *ddc1Δ* cells–deletion of *NRM1* does not rescue HU sensitivity resulting from DDC1 deletion (**Fig 5A**).

Flow cytometry shows that asynchronous *dpb2-103* cells have perturbed cell cycle, i. e. lower 1C DNA content and slow progression through S-phase (**Figs 2 and 5B**). Moreover, light scattering measurements indicate that *dpb2-103* cells are larger than wild-type cells (**Fig 5C**). This observation is confirmed by microscopic observations of *dpb2-103* cells (**S4 Fig**). Deletion of *NRM1* in *dpb2-103* cells partially suppressed these effects: the DNA content in *dpb2-103 nrm1Δ* cells shows higher 1C DNA content and lower proportion of S-phase cells (**Fig 5B**). Moreover, forward scatter (FSC) measurement indicates a decrease in cell size of *dpb2-103* mutants after *NRM1* deletion (**Fig 5C**). Together these results demonstrate that upregulation of MBF G1/S transition genes rescues several *dpb2-103* phenotypes.

**Fig 6:**
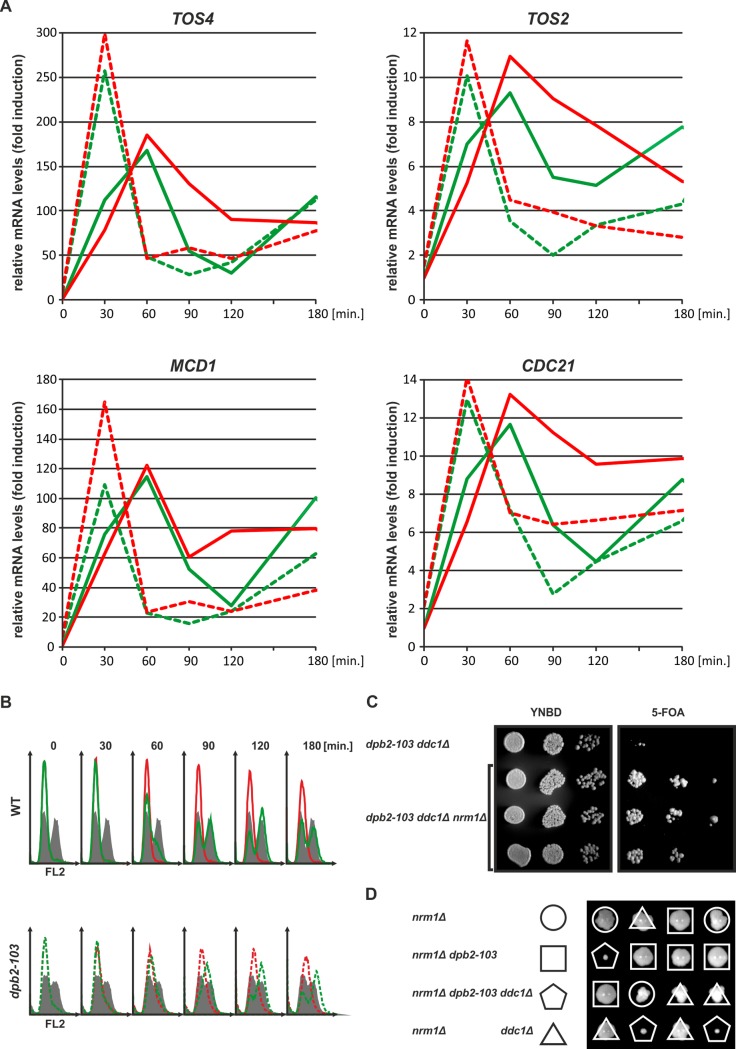
The expression of G1/S transition genes (repressed by Nrm1) from the MBF pathway is not activated in *dpb2-103* cells under replication stress. (**A)** quantitative RT-PCR analysis of *TOS2*, *TOS4*, *MCD1*, *CDC21* transcripts in wild-type (solid line) and *dpb2-103* (dashed line) cells under unperturbed growth conditions (green) or under replication stress generated by 200 mM HU (red). Transcript levels were normalized to G1-synchronized wild-type cells. Yeast cultures were synchronized in G1 and released from α-factor in YNBD or YNBD 200 mM HU. Samples were taken at time 0 (G1 arrest), 30, 60, 90, 120 and 180 minutes after release from G1 block. Standard deviations were omitted for clarity and are presented in **S1 Table**. (**B)** Flow cytometry analysis of DNA content (cell cycle progression) at 0, 30, 60, 90, 120 and 180 minutes time points in wild-type (solid line) and *dpb2-103* (dashed line) cells under unperturbed growth conditions (green) or under replication stress generated by 200 mM HU (red). **(C and D)** Deletion of *NRM1* gives viable *dpb2-103 ddc1Δ nrm1Δ* cells. **(C)** Deletion of *DDC1* in *dpb2-103* cells carrying plasmid pMJDPB2 (source of *DPB2*). Transformants were were grown exponentially and dilutions were spotted on YNBD or YNBD with 5-FOA. **(D)** Tetrad analysis of a *dpb2-103*/*DPB2*, *ddc1Δ*/*DDC1 nrm1Δ/ nrm1Δ* strain.

To support the hypothesis that activation of G1/S transition genes (the Nrm1/MBF branch) is impaired in *dpb2-103* cells under replication stress, we tested the induction of *TOS2*, *TOS4*, *MCD1 and CDC21* MCB genes repressed by Nrm1 and upregulated during S phase to promote cellular tolerance to replication stress [34]. Tos4 contains an FHA (ForkHead-associated) domain which interacts with components of the HADAC (histone deacetylase) complex involved in the response to various environmental stresses including replication stress [84]. Mcd1 is a subunit of cohesion complex involved in sister chromatide cohesion and chromosome condensation [85], Tos2 is involved in morphogenesis [86], Cdc21 is a thymidylate synthase [87]. Wild type and *dpb2-103* cells were synchronized in G1 and released into S-phase in the presence of 200 mM HU. The amount of *TOS2*, *TOS4*, *MCD1 and CDC21* RNA was normalized to wild-type untreated cells synchronized in G1 (time “0”) (**Fig 6A and S1 Table**). In parallel, cell cycle progression of these cells was monitored by flow cytometry analysis of DNA content (**Fig 6B**) demonstrating that both wild-type and *dpb2-103* cells reached the S-phase 60–90 minutes after release from G1. Interestingly, a difference between wild-type and *dpb2-103* cells, in transcription of these genes can be observed even in normal growth conditions. In wild-type cells, expression of G1/S transition genes is upregulated after release from G1 block, reaches the maximum level after 60 minutes, and decreases after 90–120 minutes. In contrast, in *dpb2-103* cells, G1/S transition transcripts are most abundant after 30 minutes of growth and reach the minimum after 60–90 minutes. More important, HU-generated replication stress induced elevated transcription of *TOS2*, *TOS4*, *MCD1 and CDC21* genes in wild-type cells but not in *dpb2-103* cells as observed at 90 and 120 minutes time points. (**Fig 6A**). We conclude there is a defect in the Nrm1 branch of the checkpoint pathway.

### Derepression of Nrm1-regulated genes rescues *dpb2-103 ddc1Δ* lethality

Positive effects of *nrm1Δ* on *dpb2-103* survival, DNA content and cell size suggest that *nrm1Δ* may restore viability of *dpb2-103 ddc1Δ cells*. Therefore, we introduced *nrm1Δ* into *dpb2-103 ddc1Δ* pMJDPB2 cells and attempted to obtain plasmid-free cells on 5-FOA. Indeed, in contrast to *dpb2-103 ddc1Δ*, we were able to obtain viable *dpb2-103 ddc1Δ nrm1Δ* cells without the plasmid carrying the gene encoding WT Dpb2 (**Fig 6C**). Similar results were obtained after tetrad dissection from a *dpb2-103/DPB2 ddc1Δ/DDC1 nrm1Δ/ nrm1Δ* diploid strain (**Fig 6D**), demonstrating that the lethal effect of the *dpb2-103 ddc1Δ* is suppressed by derepression of genes that are up regulated in checkpoint proficient cells challenged with replication stress. This strengthens our conclusion that the Nrm1 pathway is affected in *dpb2-103* and that Dpb2 and Ddc1 are involved in two separate branches of the checkpoint activation pathway.

## Discussion

Early studies of Pol ε suggested that its catalytic subunit, Pol2, is involved in replication checkpoint activation. This function has been mapped to the essential C-terminal part of the protein as shown in temperature sensitive *pol2-11* and *pol2-12* mutants, which encode subunits lacking 31 or 27 C-terminal amino acids, respectively [62,88]. Besides replication perturbations, these mutants demonstrate a subset of checkpoint deficiency phenotypes including impaired *DUN1* activation after MMS or HU treatment, low viability and elongated spindle formation after release from G1 synchronization into HU [62]. Therefore, such *pol2* mutations allow entry into mitosis despite uncompleted DNA replication. The C-terminus of Pol2 is also involved in the interaction with Dpb2, the second essential subunit of Pol ε [58,59,63]. This interaction is facilitated by cell-cycle dependent phosphorylation of Dpb2 by CDK in late G1 phase. Inactivation of phosphorylation sites of Dpb2 in the *pol2-11* strain dramatically reduces its viability, demonstrating that the Dpb2-Pol2 interaction is essential [89]. Pol2 also interacts with Mrc1 the fork-associated protein that mediates the Mec1-dependent activation of Rad53 [64]. Moreover, *mrc1Δ pol2-11* cells are inviable and overexpression of *MRC1* rescues *pol2-11* temperature sensitivity [64].

The Dpb2 subunit of Pol ε plays an essential role in maintaining the proper architecture of the replisome as it links the Pol ε with GINS and therefore the CMG helicase complex (Cdc45, Mcm2-7 and GINS) through the interaction of the Dpb2 with the Psf1 and Psf3 GINS subunits [53,55–57,90]. Therefore, it is not surprising that increased amounts of Dpb2 [22], or the four subunits of GINS [53] suppress the *pol2-11* mutation. Interestingly, both *pol2-11* and *dpb2-103* mutations impair interaction between Pol2 and Dpb2 [58,59,63]. Consequently, mutations in *DPB2* affecting proper interactions of Dpb2 with either Pol2 or GINS may disrupt the replisome integrity and influence correct activation of the DNA replication checkpoint on the leading strand. Therefore, the *dpb2-103* mutant encoding Dpb2-103 which has impaired interaction not only with the catalytic subunit Pol2 but also with Psf1 and Psf3 subunits of the GINS complex (**S2 Table**), was a good candidate for studies of DNA replication checkpoint-defective phenotypes. Using flow cytometry, we found that *dpb2-103* mutant cells, similarly to *mec1-21* mutant cells, failed to delay DNA replication after release from G1 block into MMS. (**Fig 2**). Checkpoint deficient mutant cells (*mec1* or *rad53*) have been shown previously to be unable to delay replication progression [68,91]. However, when compared to *mec1-21*, *dpb2-103* cells under MMS treatment progress through the S phase slowly and hardly reach the G2 phase, suggesting possible residual checkpoint activation (**Fig 2**). Another agent that induces replication stress, HU, slows DNA replication progression due to nucleotide depletion [8] resulting in elongating the time of origin firing [92]. Our flow cytometry experiments with G1 synchronized cells released into HU confirm that wild-type cells remain in early S even after 240 minutes. In contrast, the *dpb2-103* mutant released from G1 synchronization into HU progressed through the S phase, albeit slowly, likely due to insufficient nucleotide precursors (**Fig 2**). One can therefore speculate that S phase progression of this mutant under HU treatment results from inefficient delay of DNA replication combined with alleviation of nucleotide depletion by the slight induction of *RNR3* expression (**Fig 4B**). This hypothesis is reinforced by the observation that when compared to the wild type strain, *dpb2-103* cells progress very slowly through unperturbed S phase (**Fig 2**).

Checkpoint-deficient cells such as *mec1* mutants are also unable to resume DNA synthesis after transient HU treatment (**S1 Fig**) [20,31]. However, in our experiments, *dpb2-103* cells resume DNA replication after the HU-generated block is removed in both permissive and restrictive temperature (**S1 Fig**). This observation explains why *dpb2-103* cells are less sensitive to these drugs when compared to the *mec1* mutant (**Fig 1**).

We also observed that similarly to *pol2-11* and *pol2-12* mutants [88] untreated *dpb2-103* cells are larger, (**Fig 5C**), and that DNA content in asynchronous cells indicates that the relative proportion of S phase *dpb2-103* cells is higher when compared to *DPB2* cells (**Fig 5B**) demonstrating cell cycle control perturbations. However, the relative amount of cells in S phase may also be due to replication perturbations resulting from impaired interactions both within Pol ε and between Pol ε and the CMG helicase complex [59,61]. Nonetheless, these observations reinforce the conclusion that the cell cycle control is perturbed in the *dpb2-103* mutant most probably due to inefficient replication checkpoint activation.

**Fig 7:**
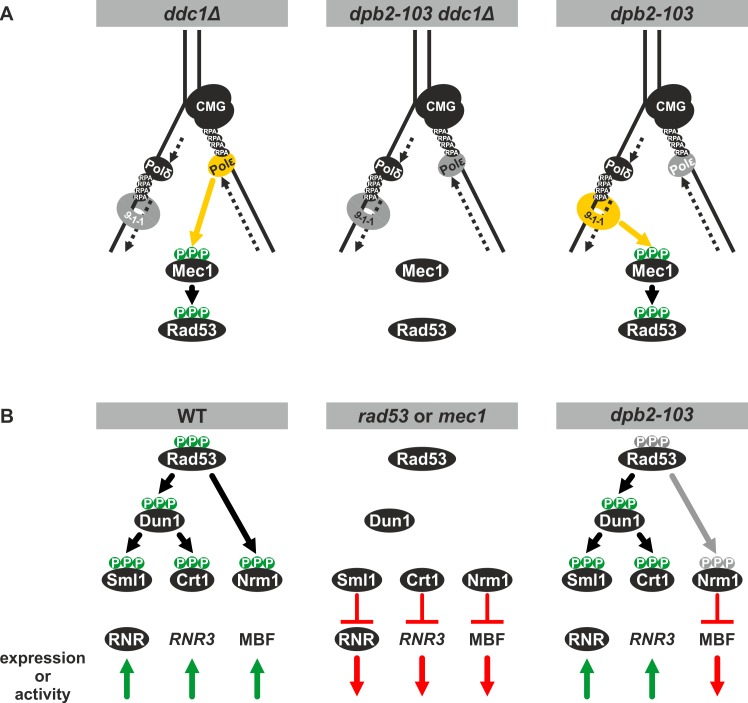
Model for replication checkpint activation and response in *dpb2-103* cells. **(A)** In *ddc1Δ* and *dpb2-103* cells replication checkpoint activation is partially impared whereas in *ddc1Δ dpb2-103* cells is abolished. (**B)** The two branches i. e. DDR (Dun1/Crt1) and CC (Nrm1/MBF) are activated in wild type cells and inactive in *rad53* or *mec1* checkpoint mutants. In *dpb2-103* cells the Nrm1/MBF branch is inactive.

Because Dpb2 is a subunit of Pol ε, the leading strand polymerase [38,41], it has been suggested that its role in sensing replication perturbations is oriented mainly toward the leading strand [20]. Then, the signal from the lagging strand would come from the 9-1–1 (Ddc1-Rad17-Mec3) checkpoint clamp which is loaded specifically at the 5’ junctions of RPA-coated ssDNA and duplex DNA [93]. The existence of two parallel leading and lagging strand-specific checkpoint activation pathways would explain partial checkpoint activation in the *pol2* mutants, the *dpb2-103* or the *ddc1Δ* cells. Flow cytometry analysis of DNA content in *ddc1Δ* cells treated with MMS demonstrated progression through S phase similar to that observed in our study for *dpb2-103* cells [18], although none of these mutants abolishes Rad53 phosphorylation in response to MMS or HU treatment [20,71] **(S3 Fig)**. Consequently, it was suggested that in *S*. *cerevisiae ddc1Δ* cells the partial checkpoint activation is mediated by Dpb11 recruited to the replication fork by Pol ε in a 9-1-1 independent manner [20,25]. Therefore, it is not surprising that our experiments showed that the double *dpb2-103 ddc1Δ* mutant is lethal (**Fig 3**), supporting a model of separate sensing of replication stress on the two DNA strands and points out the involvement of Dpb2 in this process. The interpretation that the synthetic lethality of *dpb2-103* and *ddc1Δ* results from the fact that replication defects in *dpb2-103* cells can only be bypassed by a proficient replication checkpoint is also possible. However, given that the *ddc1Δ* mutant is only partially impaired in checkpoint activation and even combined with the *dpb11-1* mutation retains low Rad53 activity that prevent replication fork breakdown this would not explain the synthetic lethality of *dpb2-103* and *ddc1Δ*. Therefore, we favor the hypothesis of separation of replication problems sensing on leading and lagging strands (**Fig 7A**).

*SML1* deletion can rescue *mec1Δ* or *rad53Δ* lethality, although it cannot restore checkpoint activation. However, after tetrad dissection of a heterozygous triple mutant we obtained colonies of very sick *dpb2-103 ddc1Δ sml1Δ* strains (**S2 Fig**), what is in accordance with our observation that the Dun1/Crt1 pathway, which regulates Sml1 degradation is properly activated in *dpb2-103* cells. It also demonstrates that the lethal effect of *mec1Δ* or *rad53Δ* mutations (rescued by *sml1Δ*) results from replication checkpoint activation/execution defects other than those occurring in *dpb2-103 ddc1Δ* cells.

The involvement of Pol ε and Pol δ in the majority of replication of the leading and lagging DNA strands, respectively, is very well documented. However, it has been proposed recently, that Pol δ is the major replicase of both DNA strands [94]. However, mutation rate data obtained using Pol2 and Pol3 mutants that incorporate unique strand specific substitutions and studies of ribonucleotide incorporation into DNA by Pol ε and Pol δ as well as DNA *in vitro* studies [37,40,95,96] strongly support the model in which Pol ε acts as the major leading strand DNA polymerase. Moreover, the model in which Pol δ is the major replicase of both strands still locates Pol ε in association with the CMG complex on the leading strand with a role in correcting replication errors and proofreading rNMPs [94].

The replication checkpoint activation results in phosphorylation of the Rad53 effector kinase and subsequent cellular response to DNA synthesis problems. The best analyzed branch of this response is the Rad53-Dun1-dependent upregulation of dNTP pool [33,75] which normally limits DNA replication, but is upregulated 6- to 8-fold under replication stress to promote fork progression [8,97]. Interestingly, although the *dpb2-103* mutant is impaired in correct response to replication stress, we detected Rad53 phosphorylation (**S3 Fig**), degradation of the RNR inhibitors Sml1 and induction of expression of the *RNR3* and *HUG1* genes (**Fig 4**). We can therefore conclude that *dpb2-103* cells activate the Dun1-Crt1 branch of replication stress response correctly (**Fig 7B**). This suggests that Pol ε checkpoint mutants are partially proficient in activation of first steps of replication stress response although with modifications in the pattern of Rad53 phosphorylation. Such incomplete phosphorylation may be undetectable in the gel retardation assay of Rad53 which has been shown to contain multiple phosphorylation sites [98–100]. Alternatively, the Pol ε signal may act downstream of or independently of Rad53 in checkpoint activation. Importantly, these observations rule out the possibility that inefficiency of replication checkpoint activation in *dpb2-103* cells results from the fact that the number of affected origins is not high enough to reach an activation threshold as demonstrated for the *orc2-1* mutant defective in initiation of DNA replication and Rad53 phosphorylation under MMS treatment [16].

The results of our analysis of the second branch of Rad53-dependent response to replication stress, the Nrm1/MBF pathway, clarify the checkpoint-deficiency phenotypes of the *dpb2-103* mutant. Rad53-dependent phosphorylation of the Nrm1 corepressor of MBF genes prevents its binding to MBF promoters in response to the S phase checkpoint [80] to activate expression of many genes involved in the replication stress response [36]. We found that *NRM1* deletion suppresses the MMS and HU sensitivity of *dpb2-103* cells (**Fig 5A**) and that *dpb2-103 nrm1Δ* cells partially rescue their cell size as well as their DNA content, demonstrating proficient progression through S phase (**Fig 5B and 5C**). We also tested *nrm1Δ* dependent checkpoint-deficiency phenotypes rescue in *pol2-12* cells. Our flow cytometry analysis shows, that the *pol2-12* mutant demonstrates a defect in S-phase progression (although less severe when compared to *dpb2-103* cells), and that deletion of *NRM1* restores proper DNA content (**S5 Fig**). Moreover, *NRM1* deletion alleviates *pol2-12* HU and temperature sensitivity (**S5 Fig**). These results suggest that the *dpb2-103* and *pol2-12* mutations in Pol ε may similarly affect the replication stress response.

The observed *nrm1Δ* dependent rescue of *dpb2-103* phenotypes during unperturbed growth can be explained by upregulation of Nrm1-regulated genes at the G1/S transition, which are prematurely downregulated in *dpb2-103* cells, when compared to the wild-type cells (**Fig 6**). Moreover, we observed that expression of Nrm1-repressed genes *TOS2*, *TOS4*, *MCD1*, *CDC21*, which is upregulated in wild-type cells even after 90–120 minutes in response to replication stress, remain uninduced in *dpb2-103* cells (**Fig 6**). Finally, the lack of Nrm1 repressor (*nrm1Δ*) partially rescued the synthetic lethality of *dpb2-103 ddc1Δ* cells; a similar lethality bypass by *nrm1Δ* has been observed for *rad53Δ* and *mec1Δ* mutants [78,79]. This demonstrates that checkpoint deficiencies in *dpb2-103* cells are due mainly to impaired derepression of Nrm1-regulated genes (**Fig 7B**). However, uncovering of the mechanism of Dpb2-dependent derepression of the Nrm1 branch of the replication stress response needs further investigation.

The question remains whether the failure of *dpb2-103* mutant to fully activate the replication checkpoint results from direct involvement of the Dpb2 in replication stress sensing / activation, protein stability changes, impaired phosphorylation or from defects in Pol ε association within the replisome. Indeed, mutations in *dpb2-103* partially impair interaction of Dpb2 with the catalytic subunit Pol2 of Pol ε and strongly impair its interaction with the Psf1 subunit of GINS. However, it would be expected that the destabilization of Pol ε in the replication fork and possibly dissociation would induce replication checkpoint activation rather than abolish it. Therefore, we favor the hypothesis of direct involvement of Dpb2 in the replication stress response, which still needs further investigation.

## Materials and Methods

### Yeast strains, media and growth conditions

*S*. *cerevisiae* strains listed in **Table 1**. were grown in standard media [101] [102]. When nutrition selection was not required yeast complete medium YPD (1% bacto-yeast extract, 2% bacto-peptone, 2% glucose liquid or solidified with 2% bacto-agar) was used. Yeast transformants were selected on YPD supplemented with appropriate antibiotics (Hygromycin B 300 μ^-ml^ or Nourseothricin 100 μg^-ml^). When necessary yeasts were selected for prototrophy on YNBD minimal medium (0.67% yeast nitrogen base without amino acids, 2% glucose, liquid or solidified with 2% bacto-agar) supplemented with appropriate amino acids and nucleotides. For selection of *URA3*-plasmid-free cells, YNBD medium supplemented with 1 mg/ml 5-fluoroorotic acid (5-FOA) was used [103]. *Escherichia coli* DH5α (F^-^, *gyrA96*, *recA1*, *relA1*, *endA1*, *thi1*, *hsdR17*, *supE44*, *deoR*, *Δ*(lacZYA-argF)U169, [φ80Δ (*lacZ*)M15]) cells were grown routinely at 37°C in L broth–liquid or solidified with 1.5% agar and supplemented when needed with ampicillin (100 μg^-ml^).

**Table 1:** Yeast strains used in this work.

Strain	Genotype	Source
SC228	*MATa CAN1 his7-2 leu2-Δ*::*kanMX4 ura3-Δ trp1-289 ade2-1 lys2-ΔGG2899-2900 DPB2*	[58]
SC232	*MATa CAN1 his7-2 leu2-Δ*::*kanMX4 ura3-Δ trp1-289 ade2-1 lys2-ΔGG2899-2900 dpb2-103*	[58]
SC765	*MATa CAN1 his7-2 leu2-Δ*::*hisG ura3-Δ trp1-289 ade2-1 lys2-ΔGG2899-2900*	[56]
Y306	*MATa can1-100 ade2-1 his3-11*,*15 leu2-3*,*112 trp1-1 ura3-1 mec1-21*	[9,108]
Y445MD	*MATa CAN1 his7-2 leu2-Δ*::*kanMX4 ura3-Δ trp1-289 ade2-1 lys2-ΔGG2899-2900 DPB2 ddc1-Δ*::*HPH*	This work
Y446MD	*MATa CAN1 his7-2 leu2-Δ*::*kanMX4 ura3-Δ trp1-289 ade2-1 lys2-ΔGG2899-2900 dpb2-10 3 ddc1-Δ*::*HPH* pMJDPB2	This work
Y447MD	*MATα CAN1 his7-2 leu2-Δ*::*hisG ura3-Δ trp1-289 ade2-1 lys2-ΔGG2899-2900*	This work
Y448MD	*MATα CAN1 his7-2 leu2-Δ*::*hisG ura3-Δ trp1-289 ade2-1 lys2-ΔGG2899-2900 ddc1-Δ*::*HPH*	This work
Y449MD	*MATa /MATα CAN1/CAN1 his7-2/his7-2 leu2-Δ*::*kanMX4/leu2-Δ*::*hisG ura3-Δ/ura3-Δ trp1-289/trp1-289 ade2-1/ade2-1 lys2-ΔGG2899-2900/lys2-ΔGG2899-2900 DPB2/ dpb2-103 DDC1/ddc1-Δ*::*HPH*	This work
Y450MD	*MATa CAN1 his7-2 leu2-Δ*::*kanMX4 ura3-Δ trp1-289 ade2-1 lys2-ΔGG2899-2900 dpb2-103 sml1-Δ*::*NAT1*	This work
Y451MD	*MATa /MATα CAN1/CAN1 his7-2/his7-2 leu2-Δ*::*kanMX4/leu2-Δ*::*hisG ura3-Δ/ura3-Δ trp1-289/trp1-289 ade2-1/ade2-1 lys2-ΔGG2899-2900/lys2-ΔGG2899-2900 DPB2/ dpb2-103 DDC1/ddc1-Δ*::*HPH SML1/sml1-Δ*::*NAT1*	This work
Y452MD	*MATa CAN1 his7-2 leu2-Δ*::*kanMX4 ura3-Δ trp1-289 ade2-1 lys2-ΔGG2899-2900 DPB2 nrm1-Δ*::*NAT1*	This work
Y453MD	*MATa CAN1 his7-2 leu2-Δ*::*kanMX4 ura3-Δ trp1-289 ade2-1 lys2-ΔGG2899-2900 dpb2-103 nrm1-Δ*::*NAT1*	This work
Y454MD	*MATa CAN1 his7-2 leu2-Δ*::*kanMX4 ura3-Δ trp1-289 ade2-1 lys2-ΔGG2899-2900 dpb2-10 3 ddc1-Δ*::*HPH nrm1-Δ*::*NAT1* pMJDPB2	This work
Y455MD	*MATa CAN1 his7-2 leu2-Δ*::*kanMX4 ura3-Δ trp1-289 ade2-1 lys2-ΔGG2899-2900 dpb2-10 3 ddc1-Δ*::*HPH nrm1-Δ*::*NAT1*	This work
Y458MD	*MATa /MATα CAN1/CAN1 his7-2/his7-2 leu2-Δ*::*kanMX4/leu2-Δ*::*hisG ura3-Δ/ura3-Δ trp1-289/trp1-289 ade2-1/ade2-1 lys2-ΔGG2899-2900/lys2-ΔGG2899-2900 DPB2/ dpb2-103 DDC1/ddc1-Δ*::*HPH nrm11-Δ*::*NAT1/nrm11-Δ*::*NAT1*	This work
Y459MD	*MATa CAN1 his7-2 leu2-Δ*::*kanMX4 ura3-Δ trp1-289 ade2-1 lys2-ΔGG2899-2900 DPB2 pol2-12*	This work
Y460MD	*MATa CAN1 his7-2 leu2-Δ*::*kanMX4 ura3-Δ trp1-289 ade2-1 lys2-ΔGG2899-2900 DPB2 nrm1-Δ*::*NAT1 pol2-12*	This work

#### DNA manipulations

Yeast strains were transformed using the LiAc/ssDNA/PEG method [104]. Total yeast DNA was isolated using the Genomic Mini AX Yeast Spin kit (A&A Biotechnology, Gdansk, POLAND). *E*. *coli* cells were transformed as previously described [105] and bacterial plasmids were isolated using the Plasmid mini kit (A&A Biotechnology, Gdansk, POLAND).

#### Construction of yeast strains

Deletions of *DDC1*, *SML1* or *NRM1* were performed by replacement of their coding regions by PCR-amplified *HPH* or *NAT1* cassettes obtained with primers listed in **Table 2**. Gene replacement was verified using PCR with primers listed in **Table 2** and sequencing. The *pol2-12* mutation was introduced as previously described [106] and verified using primers POL2DO35 and POL2DR20 (**Table 2**).

**Table 2:** Primers used in this work.

Primer	Sequence 5’-3’
ddc1HphU	GCTTAGACATATATGTCATTTAAGGCAACTATCACCGAGTCGGGGAGATCTGTTTAGCTTGCC
ddc1HphD	TATACCCCTTGGCTTTTCTACTTGTGTTAGACCCAGCCCATCTTCTTCGAGCTCGTTTTCGACAC
nrm1-P_U	AGGCTGCGGAGAGGGCCAATTGCAGCAGCGACGTACGCATAGAGGAAAAACAACCGTTCATGAGATCTGTTTAGCTTGCC
nrm1-T_D	TATTATTATATACACAATGAAGGAGAATGAAAATGAAAGATAGAAATATTGGGGGTGGGATTCGAGCTCGTTTTCGACAC
3-sml1	TGGCGCTAGCGATATCTAGC
5-sml1	CCAAACGGGCTCCACTACC
UFddc1	GCCGCCAAAGGTAATCATAGAC
UFddc1_2	GGTGCACTCAATTTGCCGAAAG
DRddc1	GCACGCTCACCAAATTGAGAC
DRddc1_2	TAGCGTTCCGGAGTATGTAGG
DDC1UO	TCAGCAGCCGTTAACTGATTCC
DDC1DO	ACACTCTGTGGCTGGAACTC
NRM1-UF	CCGACATTGACTCACTATCC
NRM1-DR	ACTTTCAGTGCTCGTGTCTC
NRM1-UO	GCCACTGCTCCTCATTAGGG
NRM1-DO	ACATCTTTCCGCGGTGTCAG
5T-sml1	CCGTGTCAACAAGAGTGTCAAGACC
sml1UO	GTCACGGTACGCAATGTGGAAG
hph UO	ACAGACGTCGCGGTGAGTTCAG
hph DO	TCGCCGATAGTGGAAACCGACG
nat1UO	ACCGGTAAGCCGTGTCGTCAAG
nat1DO	GCTTCGTGGTCGTCTCGTACTC
POL2DO35	GGTTCCCATCTGAATGTG
POL2DR20	GGTAAAGAGGCCATTGAACC
ACT1RTup	ACCGCTGCTCAATCTTCTTC [109]
ACT1RTlo	GTAGTTTGGTCAATACCGGC [109]
RNR3F682	CAAAGAGCCCGTTCAATTCG
RNR3R539	TCCAGCTCAACGGTAGTAAC
HUG1F801	TGACGATGTCGTCCTACAAG
HUG1R935	AGACCGCCGCGACGTTCGAC
CDC21F022	GAAGGAGAAGGATCCGGTAAG
CDC21R873	GAACTCACCTGGCTCCATGTC
MCD1F215	TCGGTAGACGATTCAGTCCAGATG
MCD1R362	TCGTGCGTATCAAAGTTGCGTGAG
TOS2F961	AGTGATTCCCGAATCGTCTC
TOS2R836	ACGGGAGCCTTTGCAGAGTG
TOS4F942	AGCCTAATTGTCCCTCATCC
TOS4R802	TATTCGACCTAACGGGAGAC

### Sensitivity tests

For MMS sensitivity tests, yeast strains were grown in YPD medium until OD_**600**_ reached 0,6, harvested and resuspended in 0,9% NaCl. Appropriate dilutions were plated on YNBD medium supplemented with MMS (0%, 0,01%, 0,02% or 0,03%). Colonies were counted after 5 days incubation at 23°C. For HU sensitivity tests, yeast strains were grown in YPD medium until OD_**600**_ reached 0,6 before adding HU to 200 mM final concentration. Samples were collected at indicated time points, washed with distilled water and plated on YNBD medium. Colonies were counted after 5 days incubation at 23°C.

### α-factor synchronization and cell cycle progression analysis

Yeasts were precultured overnight in YNBD medium at 23°C and appropriately diluted in YNBD medium to grow at 23°C until OD_600_ reached 0,4. Cells were harvested, resuspended in fresh YNBD medium with the α-factor mating pheromone (4 mg/ml) and grown for 2–3 hours at 23°C. Then to release them from α-factor, cells were harvested and washed three times with water. Next they were released from G1-arrest into fresh YNBD medium and incubated at 23°C. When necessary, cells were released from G1-arrest in YNBD medium containing 0,05% MMS or 200 mM HU. Samples were taken at indicated time points and fixed in 70% ethanol.

### Flow cytometry analysis

Ethanol-fixed cells were harvested, washed and resuspended in 1 ml of sodium citrate (50 mM, pH 7,0). After brief sonication they were treated with RNaseA (0,25 mg^-ml^) at 50°C for 1 hour and with proteinase K (1 mg/ml) for another hour at 50°C. Then, samples were diluted in sodium citrate containing propidium iodide (16 μg^-ml^) and incubated overnight at 4°C. The DNA content was identified by measuring the propidium iodide fluorescence signal (FL2) using Becton Dickinson FACSCalibur and the CellQuest software (BD Bioscience). To evaluate the size of yeast cells, the forward scatter (FSC) was analyzed.

### Western-blot analysis

Yeast cells were grown until OD_600_ reached 0,4–0,6. Then, 0,05% MMS or 200 mM HU was added and cells were grown for 2 h. Cells were collected and prepared for SDS-PAGE as described previously [107]. For immunodetection, goat polyclonal anti-Rad53 antibody (sc-6749) from Santa Cruz Biotechnology), rabbit polyclonal anti-Sml1 antibody (AS10 847) from Agrisera and mouse monoclonal anti-actin antibody (MAB1501) from Millipore were used.

### RNA isolation and quantitative RT-PCR

Total RNA was isolated using the Syngen Tissue RNA Mini Kit (Syngen Biotech, POLAND) as indicated in the manufacturer’s instruction. Reverse transcription was performed using the RevertAid™ First Strand cDNA Synthesis Kit (ThermoFisher Scientific) and Real-Time PCR was done using Real-Time 2xHS-PCR Master Mix SYBR (A&A Biotechnology) and LightCycler 480 (Roche). Transcript levels were normalized to actin mRNA (*ACT1*).

## Supporting Information

S1 Fig Yeast cells with *dpb2-103* mutation can still recover from HU-induced replication stress. Yeast cultures of indicated strains were synchronized in G1 and released from α-factor in YNBD medium supplemented with 200 mM HU (red). After 90 minutes, cells were washed and released in YNBD medium in 23°C (permissive temperature for *dpb2-103* mutant) or 37°C (restrictive temperature for *dpb2-103* mutant). Samples were collected after 30 and 60 minutes.
(TIF)

S2 Fig Tetrad dissection from *dpb2-103*/*DPB2*, *ddc1Δ*/*DDC1*, *sml1Δ*/*SML1* starin gives inviable *dpb2-103 ddc1Δ* or *dpb2-103 ddc1Δ sml1 Δ* cells. (TIF)

S3 Fig Detection of Rad53 phosphorylation. Extracts from WT, *dpb2-103*, *mec1-21* or *pol2-12* yeast cells treated with 200 mM HU or 0,05% MMS were resolved by SDS-PAGE and Anti-Rad53 antibodies were used for Western-blot analyzis. Unspecific bands detected using the same antibodies were used as loading control.
(TIF)

S4 Fig Microscopic images of *DPB2* and *dpb2-103* cells. Yeasts were grown in YNBD medium to log phase.
(TIF)

S5 Fig Derepression of MBF genes partially rescues *pol2-12* phenotypes. **(A)** The abnormal progression of replication in *pol2-12* cells is rescued by *NRM1* deletion. Asynchronous cells were analysed by flow cytometry to evaluate DNA content. **(B)** Deletion of *NRM1* (coding for the MBF repressor) rescues *pol2-12* HU and temperature sensitivity. Cultures of indicated strains were grown exponentially, serially diluted and spotted on YNBD supplemented with HU and incubated at 23 or 37°C
(TIF)

S1 Table Relative transcript levels normalized to wild-type G1-synchronized cells (time”0”). (PDF)

S2 Table Protein-protein interactions between Dpb2 and GINS subunits. (PDF)
