# Supplementary figures and images for: Mutations in the Non-Catalytic Subunit Dpb2 of DNA Polymerase Epsilon Affect the Nrm1 Branch of the DNA Replication Checkpoint
Source: PLoS Genet. 2017 Jan 20;13(1):e1006572. doi: 10.1371/journal.pgen.1006572 (PMC5291541; doi:10.1371/journal.pgen.1006572)

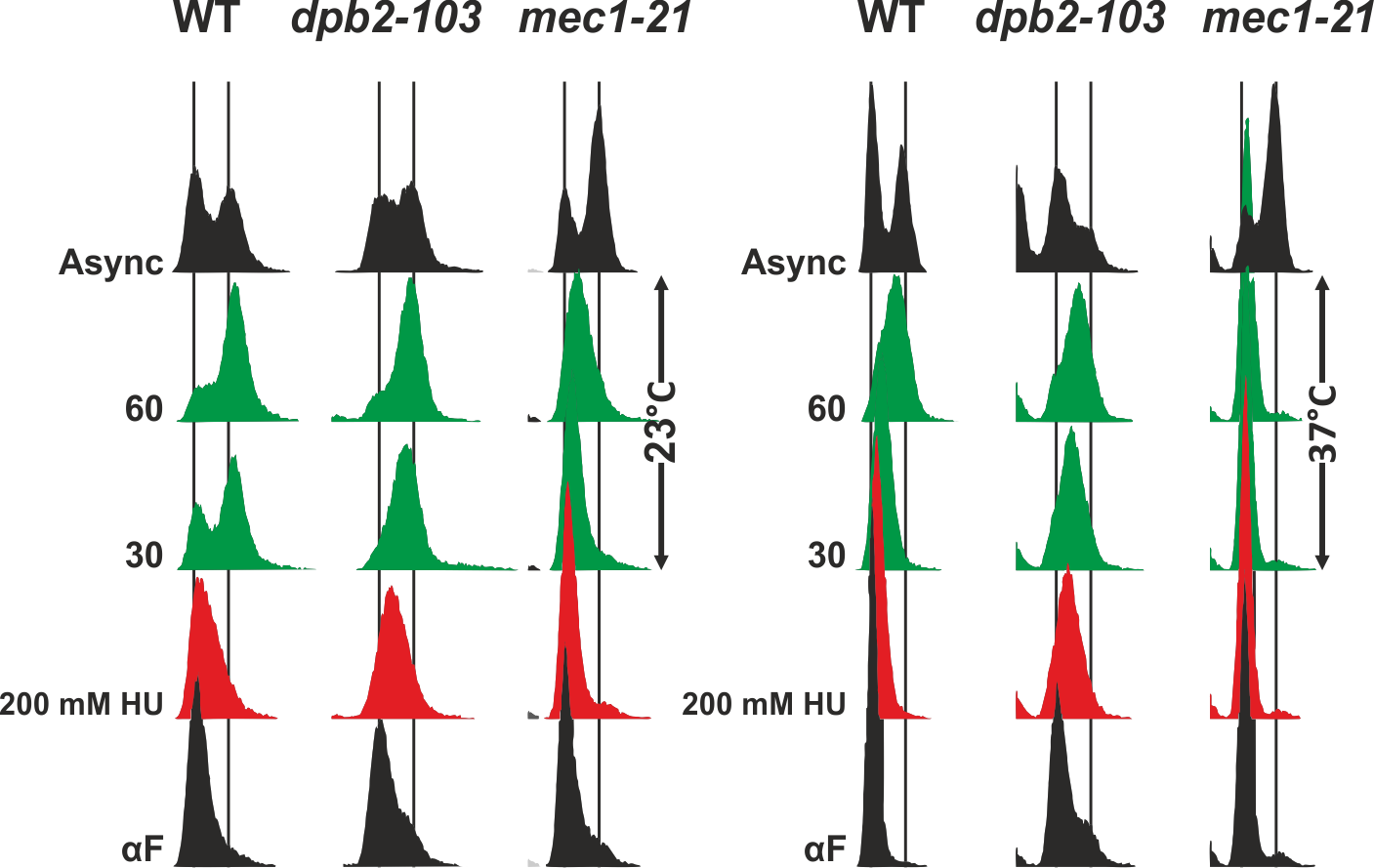

Supplement: S1 Fig — Yeast cultures of indicated strains were synchronized in G1 and released from α-factor in YNBD medium supplemented with 200 mM HU (red). After 90 minutes, cells were washed and released in YNBD medium in 23°C (permissive temperature for dpb2-103 mutant) or 37°C (restrictive temperature for dpb2-103 mutant). Samples were collected after 30 and 60 minutes. (TIF) [file pgen.1006572.s001.tif]

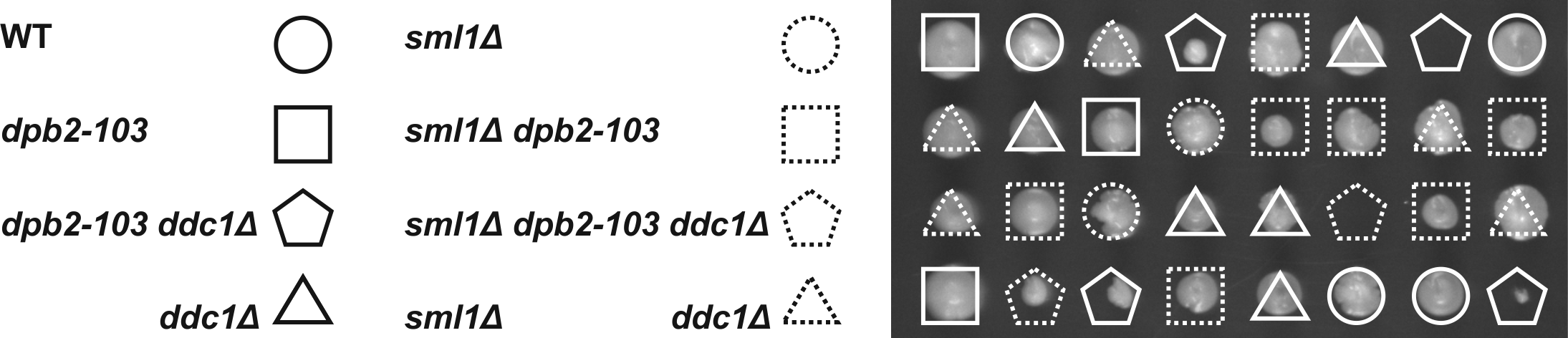

Supplement: S2 Fig — (TIF) [file pgen.1006572.s002.tif]

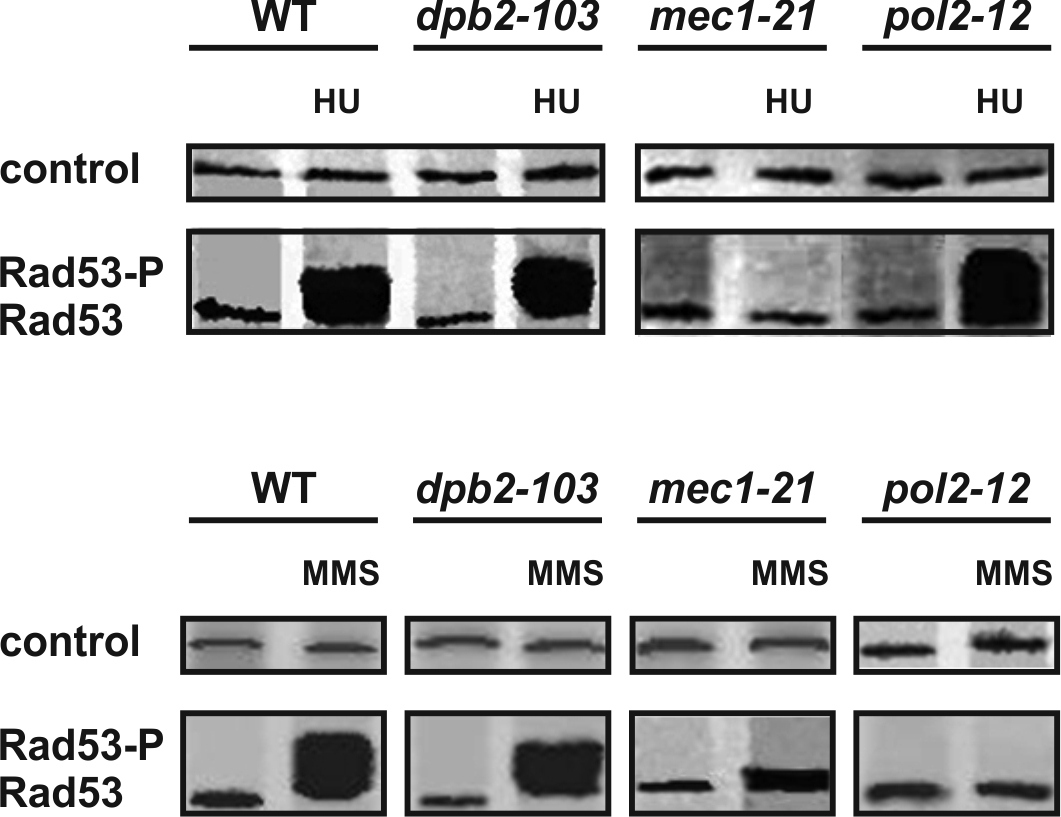

Supplement: S3 Fig — Extracts from WT, dpb2-103, mec1-21 or pol2-12 yeast cells treated with 200 mM HU or 0,05% MMS were resolved by SDS-PAGE and Anti-Rad53 antibodies were used for Western-blot analyzis. Unspecific bands detected using the same antibodies were used as loading control. (TIF) [file pgen.1006572.s003.tif]

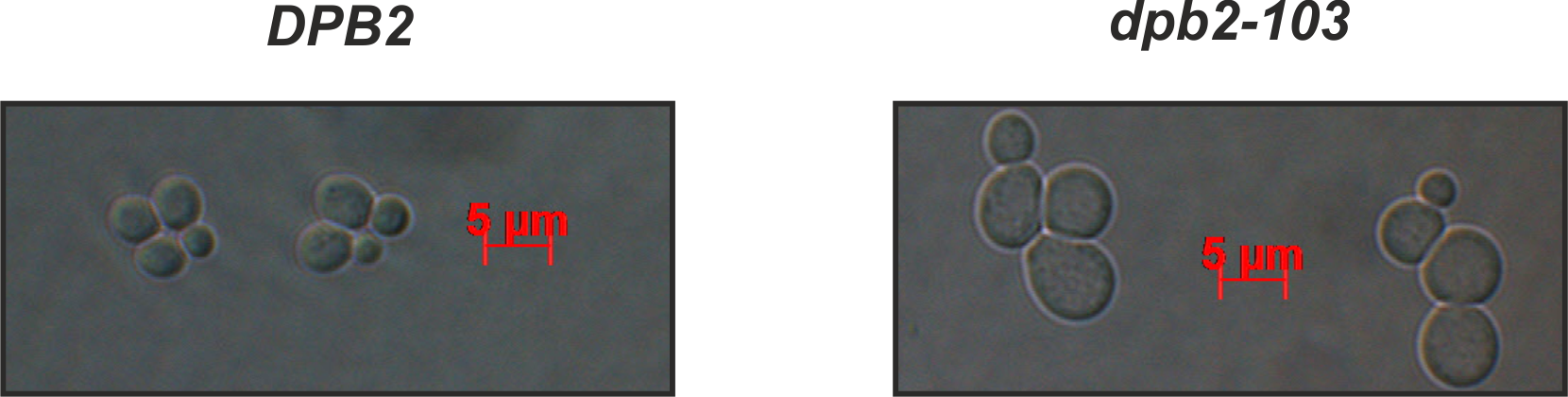

Supplement: S4 Fig — Yeasts were grown in YNBD medium to log phase. (TIF) [file pgen.1006572.s004.tif]

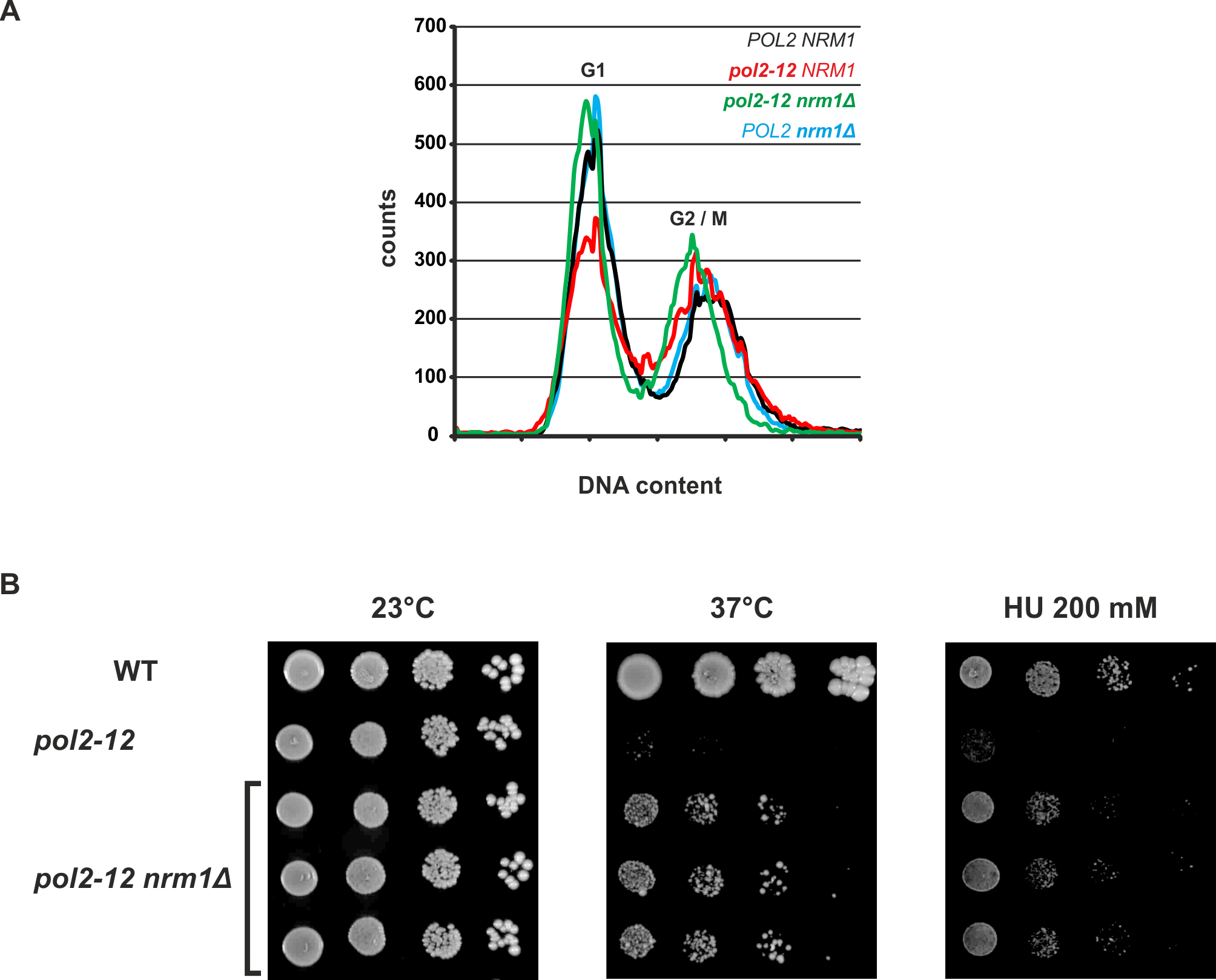

Supplement: S5 Fig — (A) The abnormal progression of replication in pol2-12 cells is rescued by NRM1 deletion. Asynchronous cells were analysed by flow cytometry to evaluate DNA content. (B) Deletion of NRM1 (coding for the MBF repressor) rescues pol2-12 HU and temperature sensitivity. Cultures of indicated strains were grown exponentially, serially diluted and spotted on YNBD supplemented with HU and incubated at 23 or 37°C (TIF) [file pgen.1006572.s005.tif]
