# Supplementary material for: Mutations in the Non-Catalytic Subunit Dpb2 of DNA Polymerase Epsilon Affect the Nrm1 Branch of the DNA Replication Checkpoint
Source: PLoS Genet. 2017 Jan 20;13(1):e1006572. doi: 10.1371/journal.pgen.1006572 (PMC5291541; doi:10.1371/journal.pgen.1006572)
Supplement: S1 Table — (PDF) [file pgen.1006572.s006.pdf]

S1 Table. Relative transcript levels normalized to wild-type G1-synchronized cells (time”0”).

		**WT**				***dpb2-103***				***mec1-21***			
**Gene**	**min.**	NT		200 mM HU		NT		200 mM HU		NT		200 mM HU	
		AV	SD	AV	SD	AV	SD	AV	SD	AV	SD	AV	SD
RNR3	0	**1,00**		**1,00**		**1,79**	0,65	**1,79**	0,65	**1,08**	0,38		
	120	**1,34**	0,71	**15,91**	2,11	**3,69**	0,63	**29,72**	9,40	**1,20**	0,41	**3,61**	0,58
	240	**3,09**	2,42	**31,36**	13,63	**6,59**	2,65	**34,39**	9,35	**0,78**	0,27	**5,02**	1,21
HUG1	0	**1,00**		**1,0**		**3,99**	1,51	**3,99**	1,51	**0,27**	0,06		
	120	**5,59**	2,80	**315,6**	75,52	**7,49**	2,46	**256,9**	92,57	**0,73**	0,15	**4,29**	0,50
	240	**5,47**	1,62	**1194,8**	506,20	**14,18**	3,07	**1257,2**	338,35	**0,60**	0,13	**16,65**	5,09
TOS2	0	**1,00**		**1,00**		**1,56**	0,49	**1,56**	0,49				
	30	**7,01**	2,70	**5,25**	2,44	**10,07**	3,72	**11,63**	5,25				
	60	**9,28**	1,44	**10,91**	2,32	**3,54**	0,92	**4,50**	1,49				
	90	**5,50**	1,40	**9,05**	2,19	**2,02**	0,65	**3,96**	1,38				
	120	**5,15**	1,05	**7,82**	1,73	**3,37**	0,81	**3,33**	0,84				
	180	**7,78**	2,92	**5,31**	2,49	**4,31**	1,76	**2,80**	0,44				
	240	**5,65**	3,60	**4,38**	3,68	**11,24**	7,55	**3,88**	1,10				
TOS4	0	**1,0**		**1,0**		**9,2**	3,5	**9,2**	3,5				
	30	**108,9**	19,1	**69,4**	19,8	**257,3**	70,0	**299,2**	100,5				
	60	**169,0**	34,2	**188,9**	44,5	**48,7**	9,6	**47,1**	23,8				
	90	**53,9**	17,1	**125,3**	38,4	**29,0**	6,7	**58,4**	16,0				
	120	**26,2**	9,6	**86,8**	19,7	**41,9**	13,9	**46,3**	18,1				
	180	**111,1**	32,0	**83,3**	27,4	**112,2**	30,6	**78,0**	23,8				
	240	**58,1**	25,1	**53,3**	17,5	**129,1**	30,7	**81,7**	29,5				
MCD1	0	**1,0**		**1,0**		**7,1**	4,0	**7,1**	4,0				
	30	**75,8**	11,8	**62,8**	15,1	**109,1**	24,9	**164,6**	33,7				
	60	**114,6**	38,4	**122,5**	30,5	**23,2**	4,5	**23,4**	8,7				
	90	**52,7**	27,3	**60,8**	13,4	**15,9**	3,7	**30,7**	7,0				
	120	**28,0**	6,8	**77,8**	20,7	**24,1**	13,5	**24,1**	9,4				
	180	**100,5**	41,3	**79,7**	30,6	**62,8**	19,1	**38,0**	8,2				
	240	**38,7**	15,3	**36,8**	11,4	**76,9**	18,5	**40,6**	8,7				
CDC21	0	**1,00**		**1,0**		**2,13**	0,53	**2,13**	0,53				
	30	**8,82**	3,54	**6,55**	2,65	**12,96**	2,68	**14,10**	2,20				
	60	**11,66**	4,39	**13,23**	3,40	**7,20**	0,88	**7,02**	2,05				
	90	**6,40**	1,77	**11,24**	2,68	**2,77**	1,18	**6,45**	0,74				
	120	**4,46**	2,23	**9,57**	2,55	**4,49**	1,16	**6,63**	1,38				
	180	**8,76**	3,07	**9,88**	2,50	**6,59**	2,68	**7,15**	2,28				
	240	**5,37**	2,60	**9,74**	4,13	**11,37**	2,42	**7,69**	4,04				
