# Supplementary material for: Mutations in the Non-Catalytic Subunit Dpb2 of DNA Polymerase Epsilon Affect the Nrm1 Branch of the DNA Replication Checkpoint
Source: PLoS Genet. 2017 Jan 20;13(1):e1006572. doi: 10.1371/journal.pgen.1006572 (PMC5291541; doi:10.1371/journal.pgen.1006572)
Supplement: S2 Table — (PDF) [file pgen.1006572.s007.pdf]

**S2 Table. Protein-protein interactions between Dpb2 and GINS subunits.**

| **Gal4 fusions** | | **β-galactosidase activity** |
|---|---|---|
| **AD** | **BD** | |
| Dpb2 | Psf1 | 304,3 (± 18) |
| Dpb2-103 | Psf1 | 3,3 (± 1,3) |
| Dpb2 | Psf3 | 70,8 (± 7,3) |
| Dpb2-103 | Psf3 | 1,4 (± 0,8) |

*DPB2* was cloned into plasmid pACT2 (Clontech) encoding the Gal4 activating domain (AD), whereas *PSF1* was cloned into plasmid pKF74, a derivative of pAS2-1 (Clontech), encoding the Gal4 DNA-binding domain (BD) [1]. The Y190 strain [2] was transformed with appropriate pairs of pKF74 and pACT2 derivatives. Protein interactions were analyzed using the *GAL4-lacZ* reporter gene using the β-galactosidase assay described previously [3].


1. Grabowska E, Wronska U, Denkiewicz M, Jaszczur M, Respondek A, Alabrudzinska M, et al. Proper functioning of the GINS complex is important for the fidelity of DNA replication in yeast. Mol Microbiol. 2014;7. doi:10.1111/mmi.12580
2. Wade Harper J. The p21 Cdk-interacting protein Cip1 is a potent inhibitor of G1 cyclin-dependent kinases. Cell. 1993;75: 805–816. doi:10.1016/0092-8674(93)90499-G
3. Rose M, Winston F HP. Methods in Yeast Genetics: A Cold Spring Harbor Laboratory Course. Cold Spring Harbor, NY: Cold Spring Harbor Laboratory Press; 1990.
